# Accelerated discovery of novel glycoside hydrolases using targeted functional profiling and selective pressure on the rumen microbiome

**DOI:** 10.1186/s40168-021-01147-1

**Published:** 2021-11-23

**Authors:** André L. A. Neves, Jiangkun Yu, Yutaka Suzuki, Marisol Baez-Magana, Elena Arutyunova, Eóin O’Hara, Tim McAllister, Kim H. Ominski, M. Joanne Lemieux, Le Luo Guan

**Affiliations:** 1 Department of Veterinary and Animal Sciences, Faculty of Health and Medical Sciences, University of Copenhagen, Grønnegårdsvej 3, DK-1870 Frederiksberg C, Denmark; 2 Department of Agricultural, Food and Nutritional Science, University of Alberta, Edmonton, Alberta T6G2P5 Canada; 3 Department of Animal Nutrition and Feed Science, College of Animal Science and Technology, Huazhong Agricultural University, Wuhan, 430070 Hubei China; 4 Research Faculty of Agriculture, Hokkaido University, Sapporo, 0608589 Japan; 5 Centro Multisciplinario de Estudios en Biotecnologia, Facultad de Veterinaria y Zootecnia Universidad Michoacana de San Nicolas de Hidalgo, 58893 Morelia, Michoacan Mexico; 6 Department of Biochemistry, Faculty of Medicine and Dentistry, University of Alberta, Edmonton, Alberta T6G 2R3 Canada; 7 Lethbridge Research Center, Agriculture and Agri-Food Canada, Lethbridge, Alberta T1J4P4 Canada; 8 Department of Animal Science & National Centre for Livestock and the Environment (NCLE), University of Manitoba, Winnipeg, MB Canada

**Keywords:** Cattle, Feed efficiency, Microbial enzymes, Rumen microbiota

## Abstract

**Background:**

Carbohydrate-active enzymes (CAZymes) form the most widespread and structurally diverse set of enzymes involved in the breakdown, biosynthesis, or modification of lignocellulose that can be found in living organisms. However, the structural diversity of CAZymes has rendered the targeted discovery of novel enzymes extremely challenging, as these proteins catalyze many different chemical reactions and are sourced by a vast array of microbes. Consequently, many uncharacterized members of CAZyme families of interest have been overlooked by current methodologies (e.g., metagenomic screening) used to discover lignocellulolytic enzymes.

**Results:**

In the present study, we combined phenotype-based selective pressure on the rumen microbiota with targeted functional profiling to guide the discovery of unknown CAZymes. In this study, we found 61 families of glycoside hydrolases (GH) (out of 182 CAZymes) from protein sequences deposited in the CAZy database—currently associated with more than 20,324 microbial genomes. Phenotype-based selective pressure on the rumen microbiome showed that lignocellulolytic bacteria (e.g., *Fibrobacter succinogenes, Butyrivibrio proteoclasticus*) and three GH families (e.g., GH11, GH13, GH45) exhibited an increased relative abundance in the rumen of feed efficient cattle when compared to their inefficient counterparts. These results paved the way for the application of targeted functional profiling to screen members of the GH11 and GH45 families against a de novo protein reference database comprised of 1184 uncharacterized enzymes, which led to the identification of 18 putative xylanases (GH11) and three putative endoglucanases (GH45). The biochemical proof of the xylanolytic activity of the newly discovered enzyme validated the computational simulations and demonstrated the stability of the most abundant xylanase.

**Conclusions:**

These findings contribute to the discovery of novel enzymes for the breakdown, biosynthesis, or modification of lignocellulose and demonstrate that the rumen microbiome is a source of promising enzyme candidates for the biotechnology industry. The combined approaches conceptualized in this study can be adapted to any microbial environment, provided that the targeted microbiome is easy to manipulate and facilitates enrichment for the microbes of interest.

Video Abstract

**Supplementary Information:**

The online version contains supplementary material available at 10.1186/s40168-021-01147-1.

## Background

The development of technologies for the effective transformation of lignocellulose into renewable, plant-based materials called biobased products is part of a global initiative to facilitate the use of biomass produced by cropping activities to address significant economic, environmental, and energy challenges [1]. Lignocellulose—comprised mainly of cellulose, hemicellulose, and lignin—is the most abundant renewable organic polymer on Earth and is the primary constituent of plant cell walls [2]. However, the highly recalcitrant nature of lignocellulosic biomass makes it refractory to efficient degradation, limiting access to the carbon sources within, and diminishing its suitability as a substrate for biobased-product synthesis [2, 3]. Enzymatic digestion of lignocellulose requires a specialized group of enzymes, known as carbohydrate-active enzymes (CAZymes), a widespread and structurally diverse group of catalysts produced by microorganisms that are constituents of various microbiomes, including that of the mammalian gut [4]. Major CAZyme families include glycoside hydrolases (GHs), polysaccharide lyases (PLs), and lytic polysaccharide monooxygenases [4, 5], with the predominant GH family widely utilized in the biotechnology and biomedical sectors [4].

Microbial communities are dynamic and can evolve the capability to produce novel CAZymes in response to changing environmental conditions (e.g., the nature of dietary substrates available for metabolism) [6]. The adaptive capacity of microbial communities facilitates the use of selective pressure—a selective advantage in the environment that causes one type of organism to grow in preference to another—through the enrichment of the microbial environment with substrates that favor the growth of microbes specialized at degrading specific substrates [7].

By analogy, it may be helpful to apply the same principle of selection pressure (e.g., utilizing an increased dietary abundance of lignocellulosic substrates) to activate and maintain a competitive fiber-degrading population (niche specialization) that expresses CAZymes with enhanced lignocellulolytic capabilities in the rumen [7, 8]. The rumen microbiome plays a fundamental role in feed efficiency of the host [9], as it provides up to 75% of the energetic requirements of ruminants through fermentation of host-indigestible plant biomass [10]. Efficient cattle are known to have a less diverse microbiome that produces more key microbial metabolites than their inefficient counterparts [11]. We speculated that this difference might be partially due to a selective advantage conferred on microbes by the rumen of efficient cattle, especially when dietary substrates (e.g., a high proportion of forage fiber) are present at sufficient concentrations to promote the development of the biochemical machinery necessary for the growth of a specialized degradative population [7]. The discovery of novel GHs within the machinery required for lignocellulose biotransformation in microbial communities has been made through metagenomic approaches [8, 12, 13], but successful application of the enzyme candidates in industrial settings has been challenging due to inconsistencies in the annotation of sequence/activity in public databases [14], as well as time-consuming and laborious nature of manual annotation. This issue is further exacerbated by the fact that individual proteins within an enzyme family can have vastly diverse functions [15, 16], complicating the identification of even closely related members.

Targeted functional profiling [17] of the active microbial community through metatranscriptomic-based approaches may overcome these limitations as it allows the compilation of a *de novo* database of marker peptides derived from reference proteins of interest. This was recently demonstrated in the discovery of a novel biomarker of host-microbial symbiosis in the human gut [16]. However, the complexity and diversity of CAZymes in microbial habitats have made targeted functional profiling of active CAZymes extremely difficult, and consequently, there remain substantial gaps in our knowledge of the functions of uncharacterized members of the CAZyme families. In this study, we used the diet and phenotype (efficient cattle) to render selection pressure to the rumen microbiome and favor the growth of microbes (and CAZymes) specialized at degrading lignocellulosic substrates. Next, targeted functional profiling of the rumen metatranscriptome was employed to discover uncharacterized enzymes in the bovine rumen according to the ecological features (e.g., abundance and frequency) exhibited by functionally distinct members of CAZyme families of interest.

## Methods

### Experimental design and sample collection

All experimental procedures described herein were approved by the Veterinary Services and the Animal Care Committee, University of Manitoba, Canada, in strict adherence to the guidelines set out by the Canadian Council on Animal Care [18]. Animals used in the current study were obtained as part of a larger project that was conducted over 2 years (*n* = 60 purebred Angus bulls/year), and details of animal management have been described previously [19]. Briefly, sixty purebred Angus bulls were randomly assigned into four pens (*n* = 15 per pen) and fed forage or grain diets over two experimental periods (Table S1). All animals were raised in confinement on the Glenlea Research Station at the University of Manitoba and maintained in the same pen throughout the experiment. For the current study, 12 (out of 15) purebred Angus Bulls (mean age of 249 ± 22 days; average body weight of 313.9 ± 32 kg at the outset of the experiment) were selected from pen 1 (year 2) because they were exclusively fed forage diets, which was used to enrich the rumen with fiber degrading microbes and their enzymes (Table S1). The experimental period lasted for 180 days, and all animals were of good health status and received an identical forage-based diet throughout the experiment. The chemical composition of the forage diet used in this study was similar to a typical commercial diet (41% neutral detergent fiber – NDF, 22% acid detergent fiber – ADF, and 22% starch) and can be found in Table S2. Feed efficiency values (calculated as feed conversion ratio (FCR); ratio of dry matter intake to average daily gain) were obtained daily using the GrowSafe® feeding system (GrowSafe Systems Ltd., Airdrie, Alberta, CA) to certify that the animals maintained their FCR ranking throughout the experiment (period 1 = 0–90 days and period 2 = 90–180 days). Three animals were removed from our study because they did not maintain the same FCR ranking throughout the experiment. Animals were then ranked according to their FCR scores and allotted into two FCR-categories with similar number of bulls in each group: (1) efficient (L-FCR; *n*=6; FCR < 6.2 kg feed consumed/kg Gain) and (2) inefficient (H-FCR; *n*=6; FCR > 6.2 kg feed consumed/kg Gain) (Data S1). Power analysis showed that we had a sufficient sample size to detect statistically significant differences between H-FCR and L-FCR groups (Fig. S1). To facilitate interrogation of rumen metatranscriptome dynamics, samples of rumen fluid were collected at 4 time points throughout the experimental period: days 0, 80, 100, and 180, using a Geishauser oral probe [20]. In this way, the effect of selection pressure on the rumen microbiota of animals with differing feed efficiencies could be investigated from day 0, which was used as the baseline to test whether the fibrolytic microbes were more abundant in the rumen of efficient cattle when compared to their inefficient counterparts. Approximately 250 ml of rumen fluid was collected at each time point, immediately snap-frozen in liquid nitrogen, and stored at −80°C for later processing.

*RNA extraction and sequencing*

Protocols for RNA isolation from rumen fluid have been previously described [21]. Briefly, 1.5 ml of TRIzol reagent (pH: 4.6; Invitrogen, Carlsbad, CA, USA) was added to approximately 200 mg of rumen sample (unthawed) in bead tubes for the subsequent RNA extraction. After homogenization, rumen samples were allowed to thaw on ice and were centrifuged at 12,000×*g* for 10 min at 4°C to obtain a pellet containing all microbial cells. The supernatant was removed and discarded, and total RNA was isolated from the pellet using a modified version of the TRIzol based acid guanidinium-phenol-chloroform (0.4 ml of chloroform—pH 7.0; 0.3 ml of isopropanol—pH 7.0; and 0.3 ml of high salt solution—pH 8.0: 1.2 M sodium acetate and 0.8 M NaCl). The Qubit 2.0 fluorimeter (Invitrogen, Carlsbad, CA, USA) and Agilent 2100 Bioanalyzer (Agilent Technologies, Santa Clara, CA, USA) were used to determine the yield and integrity of RNA samples, and only samples with an RNA integrity (RIN) value greater than 7 were used for sequencing. Next, RNA-Seq libraries were constructed using 100 ng total RNA per sample using TruSeq RNA sample prep v2 LS kit (Illumina, San Diego, CA, USA) without the mRNA enrichment step [22]. Library quality was assessed by two consecutive readings on a Qubit 2.0 fluorimeter (Invitrogen) and validated using an Agilent 2200 TapeStation (Agilent Technologies). Finally, all libraries were subjected to 2×100bp paired-end sequencing on an Illumina HiSeq 2000 platform at a commercial sequencing laboratory (McGill University and Génome Québec Innovation Centre, Montréal, QC, Canada).

### Bioinformatic and statistical analysis

Raw sequencing reads were quality-inspected using FastQC (http://www.bioinformatics.babraham.ac.uk/projects/fastqc/). Trimmomatic (v. 0.32) [23] was used to trim bases with quality scores below 20, and to remove reads shorter than 50 bp. SortMeRNA (v.1.9) [24] was used to filter out all non-mRNA transcripts, with the resulting read set used as input for contig assembly with MEGAHIT [25], using default settings. The per-base coverage depth across all contigs was calculated by mapping raw reads from each sample against the assembled contigs using BBMap (v35.92) with the parameters “kfilter=22, subfilter=15 and maxindel=80” (https://sourceforge.net/projects/bbmap/). A custom reference database consisting of all complete bacterial genomes deposited in NCBI, plus all bacterial genomes generated from ruminant feces or saliva as part of the JGI Hungate 1000 initiative, was generated for taxonomic classification of assembled contigs using Kraken (v.1.0) [26].

Gene annotation of assembled contigs was performed using MG-RAST [27]*.* Contigs were annotated using subsystems technology [28] with a maximum e-value of 10^−5^, minimum percent identity of 60, and a minimum alignment threshold of 30 bp. Assembled metatranscriptomic contigs were translated and submitted to a local version of dbCAN [29] to annotate sequences for the presence of CAZymes with an e-value threshold of 10^−5^. ShortBRED [17] was then used to determine the abundance of distinct CAZymes of interest in the metatranscriptomic dataset, with the sequences being grouped at a specified amino acid similarity threshold of 85% identity to detect non-redundant representative matches. UniRef90 was then used as the comprehensive protein reference catalog to annotate these representative peptides for each CAZyme family of interest [30]. The theoretical atomic models of the enzymes identified in the previous step were constructed using I-TASSER [31]. Multithread alignments of enzyme sequences were generated using the LOMETS meta server [32] to identify template structures from the Protein Data Bank (PDB) library, followed by reconstruction of the atomic models. Protein-ligand binding sites of the homology models were verified with the I-TASSER-associated COACH package [33]. The figures of the homology models generated by I-TASSER were prepared using Pymol (The PyMOL Molecular Graphics System, Version 2.0 Schrödinger, LLC.) and Chimera [34] (version 1.13.1). The electrostatic potential map of xylanase 1 was calculated according to the Coulomb’s Law using Chimera [34] through the following equation:

$$\varphi =\sum \kern0.5em \left[{q}_i/\kern0.5em \left(\varepsilon {d}_i\right)\right]$$

where *φ* is the electrostatic potential (which varies in space), *q* are the atomic partial charges, *d* are the distances from the atoms, and *ε* is the dielectric, representing screening by the medium or solvent.

Read counts classified by Kraken (microbial taxonomic assignment), MG-RAST (gene function), and dbCAN (CAZyme families) were subjected to differential abundance analysis (L-FCR vs. H-FCR) using edgeR under a generalized linear model [35–37]. Additionally, contrasts were used to analyze the relationship between the two FCR-categories (L-FCR vs. H-FCR) and the data collected at the 4-time points (days 0, 80, 100, 180), especially those coefficients contrasting the two-FCR groups with days 0 (baseline) and 180. All data were normalized using the trimmed mean of M-values (TMM) method as implemented in *edgeR* and calculated as the weighted mean of log-ratios between each pair of samples after excluding features with the highest counts and the largest log-fold changes. All *P* values were corrected for a false discovery rate (FDR) of 0.05 using the Benjamin-Hochberg algorithm [38], with tests inferior to the specified FDR-corrected *P* value of 0.05 considered as statistically significant. Cladograms were generated using *GraPhlAn* [39] and the heat trees were built using the *metacodeR* [40] package. All statistical analyses were performed using R 3.4.2 (R Core Team, 2017) and Python 3.6.0.

### Cloning, protein expression, and purification

The bioinformatic analysis described above resulted in the identification of a novel putative xylanase 1 protein, and the sequence encoding this polypeptide was cloned into pET43.1a vector (ligation at *Xho*1 and *Bam*H1 sites) with subtilisin protease prodomain as a tag, fused to N-terminus of protein of interest at a commercial cloning laboratory (Genscript, NJ, USA) (see the Supplementary Material to find the amino acid sequences of the enzymes). The plasmid was then transformed into chemically competent *Escherichia coli* Rosetta-gami™ 2 DE3 cells (Millipore, Ontario, Canada) and grown in Luria agar plates supplemented with 100 μg/ml of ampicillin (Amresco, Solon, OH) at 37°C. Next, a single colony was picked and transferred to 100 mL Luria broth (LB) medium with ampicillin and incubated overnight at 37°C. Afterwards, 20 mL of *E. coli* culture was inoculated into 1 L of new LB + ampicillin medium and grown at 37°C until OD_600_ reached 0.5–0.6. The protein of interest was induced with 0.4 mM IPTG and expressed for 8h at 24°C. Cells were then harvested via centrifugation at 6000×*g* for 15 min at 4°C. The cell pellets were resuspended in phosphate-buffer (pH 7.4), containing 1mM PMSF (phenylmethylsulfonyl fluoride), and lysed using Emulsiflex (Avestin, Ottawa, Canada) at a pressure of 206.8 MPa. The unbroken cells and cell debris were pelleted by centrifugation at 17,000×*g* for 30 min. The supernatant was then incubated with subtilisin resin (Profinity exact Expression Technology, Bio-RAD) for 1 h at 4 °C, and the unbounded proteins were washed away with phosphate buffer. The protein of interest was eluted by incubating the resin overnight with elution buffer (pH 7.2, 0.1M sodium phosphate and 0.1M sodium fluoride) at 4°C and then dialyzed (Spectra/Por membrane tubing, Vol/Length: 1 ml/cm) against McDougall’s buffer [41] (pH 7.0) and concentrated using 10,000 MWCO concentrators (Millipore, USA) to 0.4–1 mg/ml. The composition of the McDougall’s buffer used in this study was as follows: sodium bicarbonate, 9.80 g; sodium phosphate dibasic dehydrate, 4.62 g; potassium chloride, 0.57 g; sodium chloride, 0.47 g; magnesium sulfate heptahydrate, 0.12 g; and 4% calcium chloride, 1 mL. The concentrated protein samples were aliquoted, flash-frozen, and stored at −80°C. Protein concentration was determined by colorimetric detection and quantification of total protein using the Pierce BCA protein assay kit (Thermo-Fisher Scientific) with the bovine serum albumin as the standard. The purified protein was then visualized by SDS-PAGE gel.

### Differential scanning fluorimetry (DSF)

To investigate the effect of pH on protein stability, DFS assay of xylanase 1 in different buffers (100 mM sodium acetate buffer: pH 4.0, 5.0, 6.0; 100 mM Tricine buffer: pH 7.0, 8.0, and 9.0; McDougall’s buffer: pH 6.0, 7.0, and 8.0) was performed. Xylanase 1 in a final concentration of 5 μM was mixed with SyproOrange dye (Thermo Fisher Scientific, USA). Prior to use, the dye stock was diluted 1:50 (100X) in molecular water and used immediately while being held in darkness to reduce photobleaching. The optimal dilution of dye in the assay was determined empirically with a 5X dilution for the final assay. The thermal denaturation assay was performed in a total volume of 40 μl. All samples were run in duplicate. The thermal scan was conducted from 25 to 95°C, at 0.5°C/min (ViiA 7 Real-Time PCR System, ThermoFisher). The melting point (T_m_) was calculated by fitting the raw fluorescence data over the temperature using the Boltzmann equation in GraphPad Prism program (GraphPadPrizm 7 for Windows, GraphPad Software, USA).

### Size-exclusion chromatography

The oligomeric state and homogeneity of xylanase 1 was determined by size-exclusion chromatography on Superdex 75 (10/30) column (GE Healthcare, Canada), equilibrated with McDougall’s buffer, pH 6.0. Molar mass of the protein peak was calculated using a logarithmic interpolation of elution volumes (Ve) using a gel filtration LMW calibration kit (GE Healthcare, Pittsburgh, USA) containing (1) blue dextran 2000 (V_0_), (2) thyroglobulin (670 kDa), (3) g-globulin (158 kDa), (4) ovalbumin (44 kDa), (5) myoglobulin (17 kDa), and (6) vitamin B12 (1.3 kDa).

### Kinetic measurements

Xylanase 1 activity was determined by measuring the quantity of reducing sugars (xylose, molecular weight: 150 g/mol) released from xylan (Beechwood xylan, Megazyme) by the dinitrosalicylic acid (DNS) method [42]. Before kinetic calculations, all parameters (e.g., pH, temperature, enzyme concentration) for the assay were optimized. The minimal concentration of the enzyme that produced a linear dependence of generated product with the time was chosen, as well as the minimal time of reaction within the linear part of the curve. For kinetic measurements, xylan (substrate) was incubated at 40°C with activity buffer—McDougall’s buffer (pH 6.0) for 10 min for equilibration, and then the purified xylanase 1 was added and the reaction was performed for 10 min. The final concentration of enzyme was fixed at 0.05 μM, and the final concentration of xylan varied (0, 0.88, 1.75, 3.5, 7.0, 15.0, and 30.0 mg/ml). The total volume of reaction was 200 μl. The samples with the same concentrations of substrate but without enzyme addition were treated the same way and were used as negative controls. After adding 300 μl of DNS reagent to stop the reaction, the samples were boiled for 5 min and then incubated on ice prior to measurement of absorbance at 540 nm using a plate reader (SpectraMax M3). All reactions were performed in duplicate. The plots of the reaction velocity against the corresponding substrate concentration were fitted with Michaelis–Menten equation (*v*_0_ = *k*_cat_[E]_0_[S]_0_/([S]_0_ + *K*_M_)) using GraphPad Prism program (GraphPadPrizm 7 for Windows, GraphPad Software, USA).

### Thermal inactivation of xylanase 1 and thermodynamic analysis

A thermal inactivation assay was performed at 25, 40, 50, and 60 °C. All samples, containing 0.5μM of xylanase1 in McDougall’s buffer (pH 6.0), were incubated at the specified temperatures. A 20-μl aliquot was removed at each time point (0, 10, 20, 30, 40, 50, 60, and 70 min) and incubated on ice until the activity measurements were performed using 0.05 μM of xylanase 1 and 30 mg/ml of xylan. A non-heated enzyme was used as positive control and its activity was taken as 100%.

Enzyme inactivation over time was described as a first-order reaction according to Eq. 1:

1 $$\mathit{\ln}\kern0.5em A/{A}_0=\kern0.5em - kt$$

where *A* = activity at time *t*, *A*_*0*_ = initial activity at time zero, *k* is the inactivation rate constant at the tested temperature (min^−1^), and *t* is time (min). *k* values were calculated from linear regression analysis of the natural logarithm of residual activity versus incubation time and replotted in Arrhenius plot. Activation energy (Ea) was calculated using the slope of Arrhenius plot according to Eq. 2:

2 $$\mathit{\ln}(k)=- Ea/ RT+c$$

where *R* is the gas constant (8.314 J mol^−1^ K^−1^), *T* is the absolute temperature, and *c* is the frequency factor.

The half-life of xylanase 1 (t_1/2_ in min), defined as time after which activity is reduced to one-half of its initial value, was determined according to Eq. 3:

3 $${t}_{1/2}=\mathit{\ln}\kern0.5em (2)/k$$

The *D*-value is the time (min) needed to reduce the initial activity to 90%. It is inversely related to *k*-values and mathematically expressed in the Eq. 4:

4 $$D=\mathit{\ln}\kern0.5em (10)/k$$

The values of Gibbs free energy (ΔG°, kJ mol^−1^), enthalpy (ΔH°, kJ mol^−1^), and entropy ΔS° (J mol^−1^K^−1^) were determined as follows:

5 $$\Delta {G}^{\circ }=- RTln\kern0.5em \left( kh/k{}_bT\right)$$

6 $$\Delta {H}^{\circ }= Ea- RT$$

7 $$\Delta {S}^{\circ }=\left(\Delta {H}^{\circ }-\Delta {G}^{\circ}\right)/T$$

where *h* is the Plank constant (6.626 × 10^−34^ J s) and *k*_*b*_ is the Boltzmann constant (1.38 × 10^−23^ JK^−1^). All experiments were performed in duplicates.

## Results

*Overview of bacterial diversity, functional profiles, and CAZymes of the rumen microbiome in response to diet-based selective pressure*

**Fig. 1:**
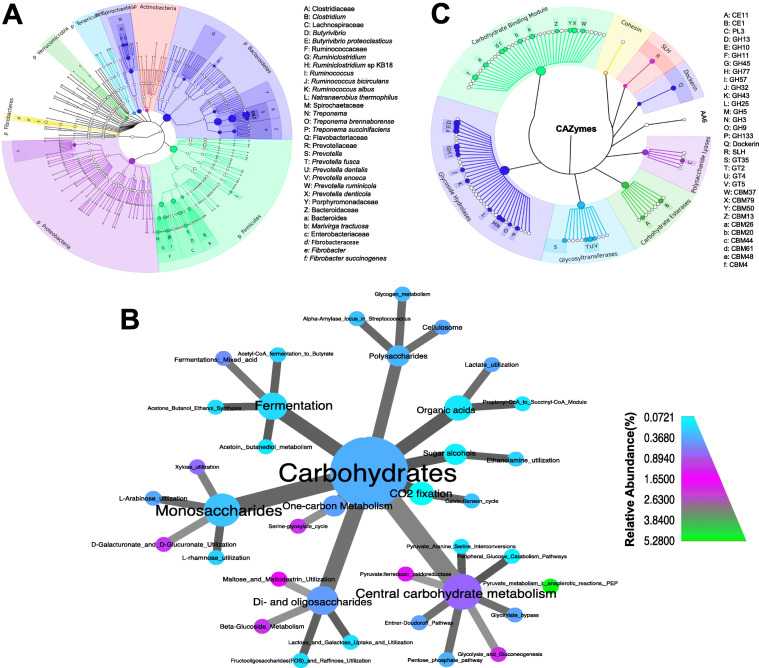
Taxonomic classification and functional capability of the rumen microbiota revealed by metatranscriptomic sequencing. **A** Cladogram showing the most abundant bacterial taxa (relative abundance ≥ 0.1% in at least half of the samples) determined by Kraken [26]. The six rings of the cladogram stand for phylum (innermost), class, order, family, genus, and species (outermost), respectively. The sizes of the circles indicate the mean average abundance of each taxon. **B** Heat tree displaying the functional capability of the rumen microbiota determined by MG-RAST [27, 28]. Each node represents the functional categories (up to three levels) and the edges determine where each node fits in the functional hierarchy. Node colors indicate the relative abundance of the functions at level 3 (functions ranged from the most detailed, level 3, to the least detailed category, level 1). **C** Cladogram showing the most abundant CAZymes determined by dbCAN [29]. The sizes of the circles indicate the mean average abundance of each CAZyme family. Cladograms were generated using *GraPhlAn* [39] and the heat trees were built using *metacodeR* [40] package. The two cladograms and the heat tree were created from an average data depicted from all animals (independent of FCR and included the four time points)

To facilitate the mining of active CAZyme families in the rumen metatranscriptome, we adopted a selective pressure approach based on the use of a forage diet—alfalfa hay and corn silage—to encourage the growth of the most competent rumen microorganisms and enzymes capable of degrading lignocellulosic substrates. RNA-sequencing of rumen fluid collected from forage-fed Angus bulls generated an average of 30M metatranscriptomic reads and 3M high quality mRNA reads, which were subsequently assembled into 7627 contigs per sample (with an average extension of 474.3 ± 26.67 bp and N50 of 462.6 ± 27.99 bp; Data S2). Approximately 51% of mRNA reads that passed quality control were successfully mapped to the assembled contigs (Data S2). Taxonomic classification of these contigs using a strategy previously developed by our group [21] resulted in the identification of 20 bacterial phyla in the rumen fluid. Among these, the majority of sequences were assigned to Bacteroidetes (45%), followed by Firmicutes (23%), Proteobacteria (14%), Spirochaetes (5.0%), Verrucomicrobia (2.3%), Actinobacteria (2.2%), Tenericutes (2.1%), and Fibrobacteres (1.4%) (Fig. 1A; Fig. S2; Data S3).

To examine the functional potential of the rumen microbiota associated with the degradation of lignocellulose, contigs were mapped against the publicly available Subsystems database using MG-RAST [27], revealing 1205 unique functions for the metatranscriptome (Data S4). Central carbohydrate metabolism (including glycolysis/gluconeogenesis, glyoxylate cycle, pyruvate metabolism and pentose phosphate pathway) and protein biosynthesis were the most abundant functional categories, representing 10% and 33% of the annotated reads, respectively (Fig. S3). In the polysaccharides- and monosaccharides-related functions, the cellulosome complex and xylose utilization systems comprised 0.39% and 0.72% of the total annotated reads, respectively (Fig. 1B; Data S4).

Assembled metatranscriptomic contigs were also aligned against the CAZyme database [4, 29] to obtain more in-depth information regarding the carbohydrate-specific enzymes in the dataset. Of the resulting 6904 alignments against the CAZyme database, transcripts assigned to glycoside hydrolases (GHs) were predominant (42.2% of the total CAZyme matches), followed by carbohydrate-binding modules (CBMs) (33.2%), glycosyltransferases (GTs) (9.8%), carbohydrate esterases (CEs) (6.6%), dockerin (2.7%), S-layer homology domains (SLHs) (2.6%), polysaccharide lyases (PLs) (2.2%), cohesin (0.5%), and auxiliary activities (AA6) (0.2%) (Fig. 1C; Data S5). Transcripts belonging to GH13 were the most abundant out of the 61 GH families identified in the rumen metatranscriptome, accounting for 6.3% of the 2914 GH hits found in the CAZyme database (Data S5). Cellulases (GH5, GH9, GH45, and GH48) and hemicellulases (GH8, GH10, GH11, GH26, GH28, GH53) transcripts also exhibited a high representation (25% of the total CAZyme matches) in the metatranscriptome (Data S5). A wide variety of non-catalytic CBMs were highly represented (2,297 hits) and predicted in interactions with various substrates such as cellulose (e.g., CBM1, CBM2, CBM3, CBM6, CBM13, CBM16, CBM44), xylan (e.g., CBM4, CBM22, CBM37), and chitin (e.g., CBM50) (Data S5). Other major classes of CAZymes abundant in our dataset were CEs (e.g., CE1, CE2, CE3, CE4, CE7, CE12) and PLs (e.g., PL1, PL9, PL11) (Data S5).

### Discovery of glycoside hydrolases through phenotype-based selective pressure on the rumen microbial community

Since there is likely a bidirectional relationship between rumen microbes and feed efficiency, the rumen microbial population in feed efficient cattle was compared with less efficient animals consuming the same diet. The FCR of the two groups of animals was statistically divergent (*P* < 0.01; Fig. S1), with L-FCR bulls consuming on average 22% less feed to achieve the same gain as H-FCR bulls (Fig. S1).

**Fig. 2:**
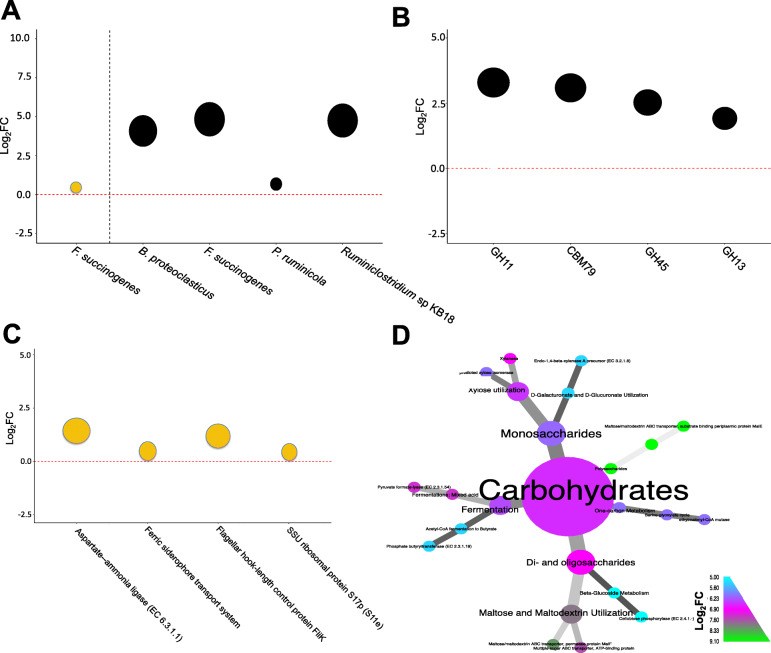
Fibrolytic bacteria and glycoside hydrolases are abundant in feed efficient cattle. **A** Log_2_-fold increase in the abundance of *F. succinogenes* in L-FCR compared to H-FCR on Day 0 (orange dot). Log_2_-fold increase in the abundance of four bacterial species on the 180th day relative to day 0 in L-FCR compared to H-FCR (dark dots). The black dashed line was drawn to separate the two time points. **B** Log_2_-fold increase in the abundance of four CAZyme families on the 180th day relative to day 0 in L-FCR compared to H-FCR (dark dots). **C** Log_2_-fold increase in the abundance of four microbial functions in L-FCR compared to H-FCR on day 0 (orange dots). **D** Heat tree showing the log_2_-fold increase in the abundance of microbial functions on the 180th day relative to day 0 in L-FCR compared to H-FCR (dark dots). Features were significant (*P* < 0.05) according to the trimmed mean of M-values (TMM) method implemented in *edgeR* [35–37]. The TMM method was used to normalize the data and minimize the log_2-_fold changes between samples. All *P* values were corrected for a false discovery rate (FDR) of 0.05 using the Benjamin-Hochberg algorithm [38], and FDR-corrected *P* values were considered as significant. In the heat tree, node colors indicate the log_2_fc in the significant (*P* < 0.05) microbial functional categories classified at level 4 by MG-RAST [27, 28]

While no difference in the overall microbial composition structure between the feed efficiency groups within the four time points was detected by Bray-Curtis dissimilarity matrices (Fig. S4), orthogonal contrasts showed that a few fibrolytic bacteria and a specific set of GHs differed between the two FCR groups within the days 0 (baseline), 80, 100, and 180. Of the 115 species present in all samples (Data S3), *Fibrobacter succinogenes* (a cellulolytic bacterium [43]) exhibited a nearly 0.5-log_2_-fold increase (*P* < 0.05) in efficient animals relative to their inefficient counterparts on day 0 (Fig. 2A). Our results also showed that the relative abundance of *F. succinogenes* and other common plant cell wall degraders (*Butyrivibrio proteoclasticus* and *Ruminiclostridium* sp KB18) exhibited > 4-log_2_-fold increase (*P* < 0.05) on the 180th day relative to day 0 in L-FCR compared to H-FCR (Fig. 2B). *Prevotella ruminicola* exhibited a 0.6-log_2_-fold increase (*P* < 0.05) on the 180th day relative to day 0 in L-FCR compared to H-FCR (Fig. 2B). It is worth mentioning that the same set of microbes listed above (*Fibrobacter succinogenes, Ruminiclostridium* sp KB18, and *Butyrivibrio proteoclasticus*), except *Prevotella ruminicola*, had already shown a significant (*P* < 0.05) log_2_-fold increase on days 80th and 100th in L-FCR relative to day 0 in H-FCR.

To further investigate the influence of feed efficiency ranking on expression of microbial CAZymes in the rumen, we then analyzed all CAZymes families in our dataset across FCR groups (Fig. 2C). We found a 1.9-log_2_-fold increase (*P* < 0.05) in the relative abundance of GH13 on the 180th day relative to day 0 in L-FCR compared to H-FCR (Fig. 2C). More importantly, we found that GH11 (endo-*β*-1,4-xylanase - EC 3.2.1.8), GH45 (endoglucanase: EC 3.2.1.4), and CBMs involved in cellulose degradation (CBM79) exhibited >2.5-log_2_-fold increase (*P* < 0.05) in their relative abundance in L-FCR compared to H-FCR on day 180 relative to day 0 (Fig. 2C). Next, we examined whether the functional potential of the microbial community was linked to the feed efficiency ranking, with the aim of finding genes involved in the degradation of lignocellulosic biomass (Figs. 2D, E). We found ammonia assimilation functions mediated by aspartate-ammonia ligases (EC: 6.3.1.1) and motor organelles, which propel the rotating flagella to enable bacteria to carry out chemotaxis [44], at a higher (*P* < 0.05) abundance in the rumen of L-FCR compared to H-FCR on day 0 (Fig. 2D). As for the degradation of monosaccharides and di- and oligosaccharides, genes related to cellulose (e.g., cellobiose phosphorylase - EC: 2.4.1.20) and xylose utilization (e.g., endo-1,4-*β*-xylanase) exhibited a >5-log_2_-fold increase (*P* < 0.05) in their abundance on day 180 relative to day 0 in L-FCR compared to H-FCR (Fig. 2E; Data S6).

### Targeted functional profiling and homology modeling of xylanases and endoglucanases

**Fig. 3:**
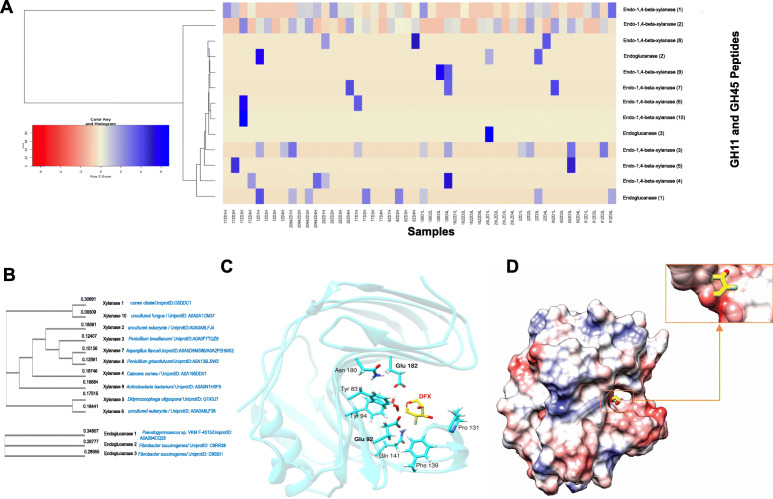
Targeted functional profiling of rumen xylanases and endoglucanases, and theoretical 3D structure of xylanase 1. **A** Heatmap showing the distribution of the most abundant members of the GH11 and GH45 families quantified according to ShortBRED [17]. **B** Phylogenetic tree of xylanases and endoglucanases detected in the rumen metatranscriptome generated by the neighbor-joining method. **C** Theoretical 3D structure of xylanase 1 showing the labeled residues involved in the binding of 1,2-Deoxy-2-Fluoro-Xylopyranose (*DFX*) (PDB ID: 1c5iA), as calculated by I-TASSER [31]. **D** Electrostatic surface analysis of xylanase 1 generated by Chimera [34], with a color spectrum that varies from electronegative (red) to electropositive (blue) values. The ligand *DFX* is bound to the enzyme at the putative active site groove with electronegative residues

To identify functionally distinct members of GH11 (xylanases) and GH45 (endoglucanases), we used the ShortBRED tool [17] to screen those families against a de novo protein reference database comprised of 1184 uncharacterized enzymes retrieved from UniProt, and then profiled their abundance and distribution in the rumen metatranscriptome. By screening 775 uncharacterized members of the family GH11 and 409 of the family GH45, we identified 18 putative xylanases (GH11) and three putative endoglucanases (GH45) in the rumen (Fig. 3A; Data S7). In this study, bacteria and eukaryotic organisms represented the major sources of the identified xylanases and endoglucanases (Fig. 3B). While only two genes were sourced from known rumen microbes such as *F. succinogenes* (100% identity; UniProt IDs: C9RR38 and D9SBI1) and uncultured rumen ciliates (UniProt ID: G5DDC1; 70.5% identity with *Epidinium caudatum;* Fig. S5), the vast majority of enzymes matched bacterial and fungal strains found in other environments (e.g., soil; UniProt IDs: A0A2P2HMK2, A0A165DD01, A0A094CQ25), highlighting the relatively poor functional annotation of the rumen microbiome (Fig. 3B).

To further investigate putative active site residues and the tertiary conformation of the identified xylanases and endoglucanases, we constructed and compared the homology models of the enzymes with known crystal structures deposited on the Protein Data Bank (PDB) using I-TASSER [31] (Figs. S6 and S7; Table S3). Of the 33 crystal structures reported for the family GH11 in the PDB and CAZyme databases, the top homology model of xylanase 1 (the most broadly distributed and abundant xylanase in our rumen metatranscriptome dataset; Fig. 3A; Data S7) showed 57% sequence identity with the crystal structure of a xylanase family 11 (PDB ID: *1h4hB*) encoded by *Bacillus agaradhaerens* (Fig. S6A and Table S3). The confidence score (C-scores) of the top model for xylanase 1 was 1.67, indicating good quality of the predicted homology model (C-score is typically in the range [−5, 2], where higher scores signify a model with high confidence). Superimposing the homology model of xylanase 1 onto the crystal structure of *Bacillus agaradhaerens* xylanase resulted in a global structural alignment score (TM-score) of 0.95 (TM-score >0.5 indicates a model of correct topology) and root-mean-square deviation of the TM-aligned residues of 0.47 Å, confirming that the model was in agreement with the crystal structure of *Bacillus agaradhaerens* xylanase.

Multiple functional annotations performed by I-TASSER in conjunction with the COACH package [33] revealed the ligand 1,2-Deoxy-2-Fluoro-Xylopyranose (*DFX*) (PDB ID: *1c5iA*) docked in the predicted substrate-binding cleft of xylanase 1 (Fig. 3C; Fig. S7; Table S3), which is a protein that exhibits a *β* jelly-roll fold typical of GH11 xylanases [45]. The surface analysis of the model using solvent accessibility prediction scores (SA) [31, 46] showed that the residues bound to *DFX* (Fig. 3C) exhibited low SA scores (ranging from “0”, buried residue, to “9”, highly exposed residue: Glu182 “1”; Tyr94 “0”; Tyr83 “0”; Glu92 “0”; Gln141 “0”; Phe139 “1”; Pro131 “1”), indicating that the active site amino acid residues were buried in the cleft or groove of the enzyme. Further analysis revealed two predicted catalytic residues located on *β* strands 9 and 14 and separated from each other by ~5.7 Å: (1) Glu92 which has a nucleophilic function and (2) Glu182 which is the proton donor (Fig. 3C)*.* The electrostatic map [34] (red—negative potential; blue—positive) showed that those predicted active site residues lied in an area of the enzyme with a negative electrostatic potential, which makes the groove a suitable active site to attract the positively charged ligand *DFX* (Fig. 3D).

### Biochemical characterization of Xylanase 1

**Fig. 4:**
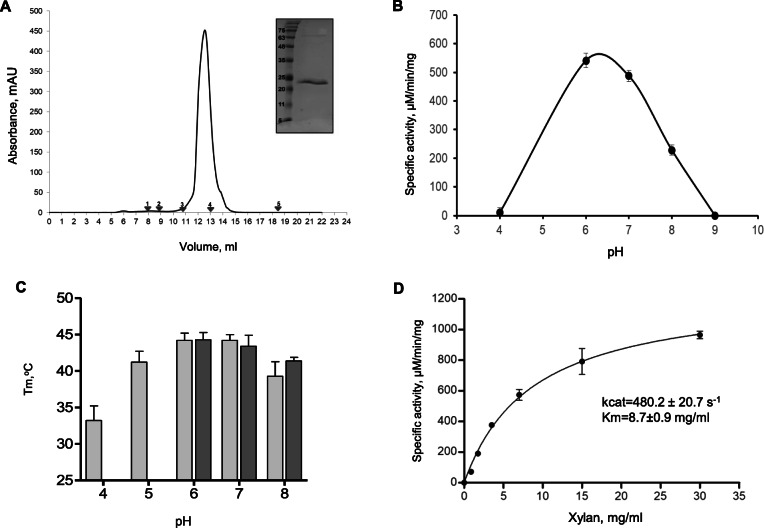
Purification and functional characterization of xylanase 1. **A** Size-exclusion chromatography of xylanase 1 on Superdex200 (10/300) column revealing the monomeric state of the enzyme. Inset: SDS-PAGE analysis of eluted xylanase 1. **B** The dependence of xylanase 1 activity on pH. **C** Melting temperatures (T_m_) of xylanase 1 determined by differential scanning fluorimetry (DSF) in different buffers (Light grey: 100 mM sodium acetate buffer: pH 4.0, 5.0, 6.0; 100 mM Tricine buffer: pH 7.0 and 8.0; Dark grey - McDougall’s buffer: pH 6.0, 7.0, and 8.0). **D** Michaelis-Menten plot of xylanase 1-mediated cleavage of Beechwood xylan. Xylan at the concentration range from 0.9 to 30 mg/ml was incubated with 0.05 μM of xylanase 1 in McDougall’s buffer (pH 6) at 40°C (*n*=3 ± standard deviation of duplicate reactions)

To confirm the lignocellulolytic activity and stability of the enzymes, we prioritized the most broadly distributed and abundant xylanase identified in the rumen metatranscriptome to validate the bioinformatic results via biochemical characterization. The purification of xylanase 1 from a protein expression vector *pET43.1a* cloned with transcripts encoding xylanase 1 and expressed in *E. coli* resulted in a highly purified enzyme with an expected molecular mass of 23 kDa (Fig. 4A). Gel-filtration analysis revealed that xylanase 1 eluted as a single homogeneous peak with a calculated molecular mass of ~ 25 kDa, suggesting that the protein exists in a monomeric state in solution (Fig. 4A).

The pH optimum of the newly identified enzyme was determined by estimating its catalytic activity at pH 4.0–9.0 using Beechwood xylan as substrate. The graph of dependence of specific activity on pH exhibited a standard bell-shaped curve with an optimum pH of 6.0, with the enzymatic activity being still high at pH 7.0 and decreasing only at pH 8.0 (Fig. 4B). To confirm the pH dependence results and validate the optimal conditions for assessing the catalytic parameters of xylanase 1, we performed a “thermal shift” assay through differential scanning fluorimetry to measure changes in the thermal denaturation temperature and identify the most stable pH and buffer conditions for the enzyme. The melting temperatures (*Tm* values) of xylanase 1 were examined at pHs ranging from 4.0 to 9.0 in widely used buffer systems, which included sodium acetate for low pHs, Tricine for high pHs, and McDougall’s buffer, a standard buffer system primarily used for assessing feed fermentation parameters in rumen studies [41]. As seen in Fig. 4C, the *Tm* values in McDougall’s buffer were similar to those observed in the Tricine buffering system, with the highest *Tm* recorded at pH 6.0. The “thermal shift” assay showed the same standard bell-shaped curve observed in the relationship between specific activity and pH dependence (Figs. 4B, C).

**Fig. 5:**
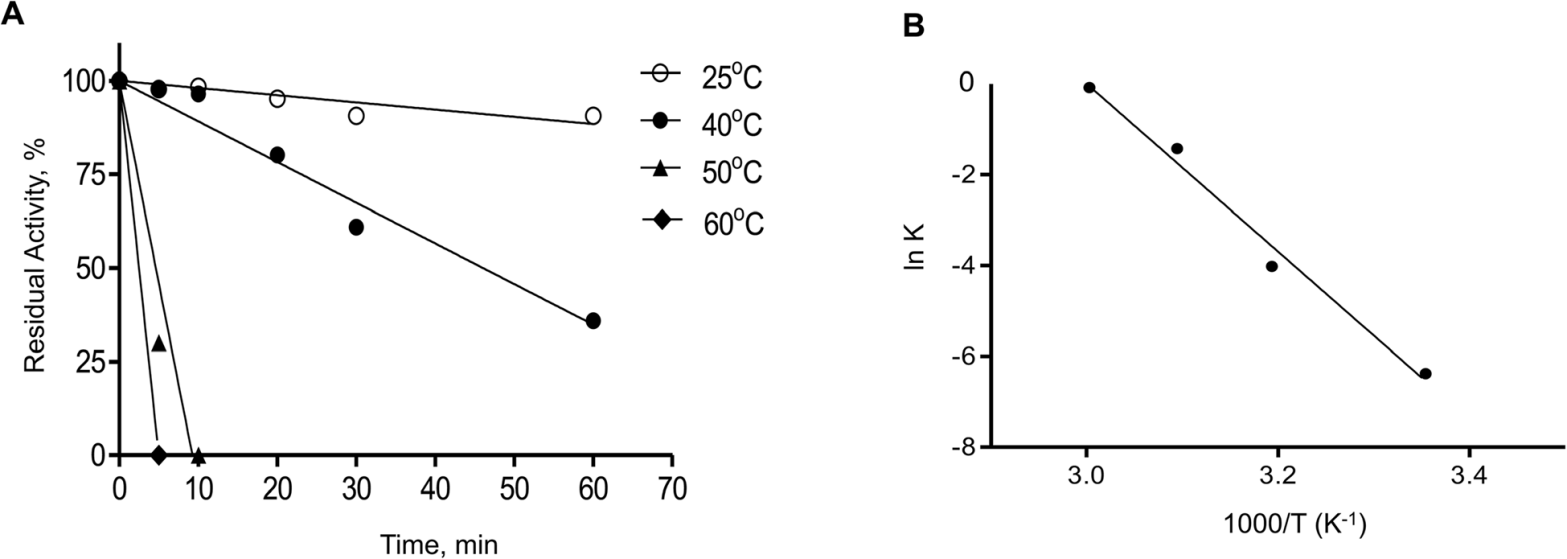
Thermal stability of xylanase 1. **A** Time course of residual activity (%) of xylanase 1 at different temperatures (*n* = 2 measurements). **B** Arrhenius plot showing the natural logarithm of the reaction rate constants (*k*) as a function of the reciprocal of the specified temperatures used to determine the apparent activation energy (*Ea*) of thermal inactivation calculated as 154.86 kJ mol^−1^

The catalytic parameters of xylanase 1 towards the cleavage of xylan were then calculated according to the Michaelis–Menten kinetics (Fig. 4D), and the cleavage rate and catalytic efficiency were 480 ± 20.7 s^−1^ and 872 ± 43.2 M^−1^s^−1^, respectively, with a *Km* of 8.7 ± 0.9 mg/ml (Fig. 4D). Next, we evaluated the inactivation kinetics and studied the thermodynamic parameters of xylanase 1. The effect of temperature on xylanase 1 stability was examined by incubating the enzyme at temperatures in the range of 25–60°C for 5–60 min (Table 1; Fig. 5). The semi-log plots of the residual activity versus heating time were linear at all temperatures studied (Fig. 5), suggesting that inactivation of xylanase 1 is a simple first-order monophasic process. Inactivation rate constants calculated from the slopes of semi-log plots for a first-order reaction showed that *Kd* values increased ~10-fold per 10 °C during the heat inactivation process, suggesting that although the increase in temperature augmented the rate of reaction it also caused the denaturation of the enzyme and its loss of functionality (Table 1). In fact, the results showed that xylanase 1 was stable at 25 °C as it lost only 10% of its activity after 1 h of incubation (*D* value of 1354 min) (Table 1; Fig. 5A). At 40 °C, 35% of its activity remained after 1 h of incubation, with a *D* value of 127 min. The loss of activity with a *D* value of 2.5 min was observed after incubating the enzyme at 60 °C (Table 1; Fig. 5A). When the dependence of *Kd* in relation to temperature was fitted through the Arrhenius equation, the results showed a simple linear fit for the reaction (Fig. 5B). The apparent activation energy (*Ea*) of thermal inactivation was 154.86 kJ mol^−1^, indicating that the transition state of the reaction displayed a relatively high energy barrier to be lowered by the enzyme.

**Table 1:** Kinetic parameters characterizing the thermal inactivation of xylanase 1

Temperature (°C)	*K*_d_ (min^−1^)	t_1/2_ (min)	D (min)	ΔH° (kJ∙mol^−1^)	ΔG° (kJ∙mol−^1^)	ΔS° (J∙mol^−1^∙K^−1^)
25	0.0017	407.73	1354.46	150.52	78.64	0.24
40	0.0180	38.51	127.92	150.40	76.58	0.24
50	0.2400	2.89	9.59	150.31	72.16	0.24
60	0.92	0.75	2.50	150.23	70.75	0.24

*K*_*d*_, inactivation rate constant; *t*_*1/2*,_ half-time (i.e., the time after which activity is reduced to one-half of the initial value); *D*, the time required to reduce enzymatic activity to 10% of its original value; *ΔH*°, activation enthalpy; *ΔG°*, activation free-energy barrier; *ΔS°*, activation entropy of thermal denaturation

The thermodynamic parameters of inactivation, including Gibbs free energy change (Δ*G*°), enthalpy change (Δ*H*°), and entropy change (Δ*S*°), were also assessed to understand the enzyme’s behavior at each step of the heat-induced denaturation process. The value of Δ*H*° was on average 150.36 kJ mol^−1^, and within the measurements error range it was independent of temperature, assuming that there was no change in the enzyme heating capacity (Table 1). The positive results for Δ*H*° at all temperatures studied indicated that the enzyme inactivation process was endothermic (Table 1), meaning that the formation of xylose monomers in the reaction required the input of additional energy from a heat source. The results also revealed that Δ*G*° values declined from 78.64 to 70.75 kJ mol^−1^ when the incubation temperature increased from 25 to 60°C, indicating protein destabilization with increasing temperature (Table 1). The fact that Δ*G*° was > 0 and *Kd* < 1 at all temperatures indicated that the reactants were favored over the products at equilibrium and so the equilibrium mixture contained more reactants than products. The Δ*S*° estimates were positive at all temperatures, indicating that there was an increase in the molecular randomness or disorder during the exposure of the enzyme to higher temperatures and that the unfolding of xylanase 1 was a rate-limiting step for thermal inactivation (Table 1). This characterization provides a strong basis for its use in degradation of lignocellulose.

## Discussion

The diverse and dynamic nature of the rumen microbiome has seen it emerge as a promising reservoir for the identification of novel microbial proteins with potent application in the biotechnology sector, most notably those involved in the hydrolysis of lignocellulosic biomass [47]. While recent studies have successfully used metagenomic-based approaches to predict and identify putative genes encoding novel CAZymes [8, 12, 13, 48], this methodology fails to capture the functional divergence of individual proteins within each family of CAZymes, as it does not consider the structural diversity among family members. To address this, the present study employed principles of microbial ecology (diet- and phenotype-based selective pressure) and adapted existing bioinformatic approaches (targeted functional profiling) to recover novel enzyme sequences of distinct GH family members in the rumen metatranscriptome of Angus bulls fed a forage diet. While targeted functional profiling has been used to examine uncharacterized enzymes found in the human gut microbiome [16], these studies have not comprehensively profiled CAZyme families in microbial communities. Similarly, although both metagenomic and metatranscriptomic screenings have been widely applied to discover CAZymes in various microbiomes [14, 49–51], to our knowledge, the use of selective pressure on the native microbial community in combination with targeted functional profiling has not been applied to quantitative metatranscriptomic analysis to identify novel enzymes.

Previous studies have shown that incubating recalcitrant substrates (as high as 51–77% NDF and 43–48% ADF) for a short time (~72 h) in the rumen of cannulated animals or in in vitro systems is a valuable strategy to identify novel CAZymes from the enriched microbial environment [12, 48]. However, our study has demonstrated that the long-term intake of relatively low NDF and ADF concentrations (41% and 21%, respectively) can still confer a selective advantage upon microbes that produce specialized fiber-degrading enzymes in feed-efficient animals. Cellulose- and hemicellulose-degrading bacteria and a specific group of CAZymes were more abundant in the rumen of efficient cattle than in their inefficient counterparts undergoing the same degree of selection pressure, highlighting the importance of individualized microbiome response within each phenotype in the capacity to express the enzymes of interest. Taken altogether, these findings provide more robust evidence of a causal relationship between the rumen microbes and feed efficiency than has previously been reported [9].

It is worth mentioning that *we also detected the abundance of the SLH domain*, which is part of a large multi-enzyme complex known as the cellulosome [52]. The presence of accessory modules (>400 hits in this study) commonly found in bacterial and fungal cellulosome-associated structures (AAs, dockerins, and cohesins) and the SLH domain, provided additional evidence of active cellulosome-mediated plant cell-wall degradation employed by rumen microorganisms (Data S5). *To the best of our knowledge, it is the first time that the SLH domain has been documented in the rumen microbiome of cattle, although it was previously characterized in the camel* *[*53*]* and muskoxen rumen [54]. Its presence in the bovine rumen lends further credence to the hypothesis that feeding animals forage diets over a prolonged period of time promotes the proliferation of microbes and enzymes involved in plant cell wall hydrolisis in the rumen microbiome.

Having characterized the presence of enzyme families which we expected to proliferate in the forage-fed rumen, we then applied a targeted functional profiling strategy to identify enzymes of unknown functions within the GH11 and GH45 families, as these families exhibited the greatest growth in the rumen of efficient versus inefficient animals (Fig. 2). Although the activities of several GHs have been investigated in the rumen, many members of the GH11 and GH45 families remain uncharacterized and are typically absent in analyses of the rumen proteome. GH11, unlike other xylanase families (e.g., GH10), comprises only endo-*β*-1,4-xylanases whose function is to cleave *β*-1,4-xylosidic bonds between xylose monomers, whereas endo-1,4-*β*-glucanases of the family GH45 play a role in the hydrolysis of the 1,4-*β*-D-glucan chain. Although all of these enzymes act on lignocellulosic substrates (xylose and cellulose) and are classified into their respective families based on sequence similarities [4], they exhibited divergent abundances in the rumen of cattle with differing feed efficiencies in the present study, indicating that they may perform distinct activities within the GH11 and GH45 families (Fig. 3A). Of the 21 novel lignocellulolytic enzymes (18 xylanases from the GH11 family, and 3 endoglucanases belonging to the GH45 family), xylanase 1 was the most abundant catalyst identified in the rumen metatranscriptome. Kinetic and thermodynamic analyses confirmed its status as a stable enzyme capable of degrading xylan. Moreover, we constructed a homology model of xylanase 1, which revealed a similar structural fold and catalytic residues as that of *1h4hB* xylanase, but it showed different rearrangements in the loops that make up the active site. Even subtle loop variations can impact substrate recognition and the catalytic activity of enzymes [55] and suffice to differentiate them from other members of the GH11 family. This highlights the critical contribution of the targeted functional profiling to the rapid divergence of as-yet-uncharacterized members in these families.

As mentioned earlier, the location of the catalytic residues (Glu92 and Glu182) in xylanase 1 confers a conformation similar to other GH11 xylanases and is entirely consistent with the catalytic apparatus of a retaining glycoside hydrolase that hydrolyzes glycosidic bonds by a double displacement mechanism [56]. In xylanase 1, the residue in close spatial proximity to Glu182 (the catalytic acid-base) was Asn180 (Fig. 3C), which is a characteristic of enzymes that function under more alkaline conditions [57]. Our experiments confirmed that xylanase 1 exhibited a pH optimum (global mean) similar to the rumen pH of forage-fed cattle (~pH 6.0–7.0) [58], and thus, it is likely that this enzyme plays a significant role in the digestion of xylan in the rumen. Under optimum pH and temperature conditions, xylanase 1 displayed high catalytic activity against Beechwood xylan (*Kcat* was > 10-fold higher) compared to other rumen GH11 xylanases reported to date [59]. As a major constituent of forage-rich diets, effective xylan degradation is a key function of the rumen microbiome [43] (regardless of FCR status), and this highlights the finding that xylanase 1 was detected in all animals, indicating that its role as a xylan degrader is conserved across individuals and feed efficiencies.

While our approach effectively discovered novel endoxylanases and endoglucanases in the rumen and validated a candidate with the potential to break down xylan, limitations exist in our study, such as the potential mapping of reads/contigs to unrelated enzymes with a better score if they were part of the database. As future directions, we propose to use a new algorithm called CUPP (Conserved Unique Peptide Patterns) [60, 61] in conjunction with dbCAN to identify the CAZymes in the rumen. CUPP has produced F-scores, sensitivity, and precisions of family and subfamily annotations that match or represent an improvement compared to state-of-the-art tools like dbCAN. In addition to grouping the enzymes at a level lower than protein families and/or subfamilies, CUPP has a database (library of conserved peptides) curated and updated every year. The implementation of the CUPP pipeline in the next stage of this research will definitively strengthen the power of our combined approach (selective pressure + targeted functional profiling) to identify uncharacterized enzymes in the rumen.

## Conclusions

The discovery of novel lignocellulolytic enzymes is of great priority, having application in both the biotechnology sector and as a means of understanding, and potentially improving, the efficient degradation of plant biomass in the rumen. Unfortunately, a considerable amount of agricultural residuals are underutilized due to the lack of an effective enzymatic system to degrade lignocellulose and release its constituent sugars for fermentation. The current study highlights the usefulness of combining selective pressure on the native rumen microbial community with targeted functional profiling using well-established algorithms [17, 26, 27, 29]. This approach revealed not only the diversity of bacteria and genes associated with efficient plant cell wall digestion but also facilitated the characterization of novel CAZyme family members, which may be critical in feed degradation. Applying this strategy and its underlying concepts in microbial ecology, nutrition, and bioinformatics, we discovered several previously uncharacterized xylanases and endoglucanases. The demonstration of the xylanolytic capacity of the most abundant and conserved member of the GH11 family (xylanase 1) validates the power of this strategy in discovering lignocellulolytic enzymes from the rumen microbiome. However, the structural basis of these new enzymes must be investigated in more detail to consolidate their status as suitable candidates for the industrial enzyme market. Notwithstanding, these findings may have many applications outside of animal agriculture for the discovery and characterization of novel CAZymes, particularly in the identification of novel microbial enzymes for use in the biotechnology sector. It may be adapted to any microbial environment for the discovery of CAZymes of interest, provided that the targeted microbiome is easy to manipulate and facilitates enrichment for the microbes of interest. This approach will encourage the evolution of microbes specialized at digesting lignocellulose, and it is likely that in the future, many more lignocellulolytic enzymes with high catalytic efficiency will be discovered as knowledge of the precise factors which drive microbial community shifts improves. Application of this approach on a larger scale may allow further discovery of novel CAZymes within the rumen and other host-associated microbiomes, which may have critical functions in feed digestion and host health, as well as applications in the biotechnology industry.

## Supplementary Information

**Additional file 1: Fig. S1**. Feed conversion ratio (FCR) and average daily gain (ADG) in bulls fed forage diets and divided into two groups of feed efficiency: 1) efficient or low feed conversion rate (L-FCR) and 2) inefficient or high-FCR (H-FCR). **Fig. S2**. Top 10 most abundant phyla detected in the rumen metatranscriptome of cattle fed forage-based diets. **Fig. S3**. Top 10 most abundant Level 2 functions detected in the rumen metatranscriptome of cattle fed forage-based diets. **Fig. S4**. Analysis of multivariate homogeneity of group dispersions between H-FCR and L-FCR. **Fig. S5**. Protozoa and fungi species identified in the rumen metatranscriptome according to the feed efficiency groups. **Fig. S6**. (A) Homology model of xylanases identified in the rumen microbiome aligns well with crystal structures of xylanases of the GH11 family. **Fig. S7**. (A) Homology model of xylanases and endoglucanases identified in the rumen microbiome bound to the predicted ligands. **Table S1**. Ingredient and chemical composition of the forage diet used in this study. Table S2. Details of the homology models predicted by I-TASSER. Amino acid sequences of the ten most abundant GH11 and GH45 enzymes identified in the rumen metatranscriptome. **Table S3.** Details of the homology models predicted by I-TASSER [3, 4].

**Additional file 2: Data S1**. FCR ranking of the experimental animals.

**Additional file 3: Data S2**. Assembly statistics of the samples investigated in this study.

**Additional file 4: Data S3**. Bacterial composition (phyla, family, genera, and species) detected in the rumen metatranscriptome of forage-fed bulls.

**Additional file 5: Data S4**. Microbial functions (levels 1, 2, 3 and 4) detected in the rumen metatranscriptome of forage-fed bulls.

**Additional file 6: Data S5**. CAZyme families identified in this study.

**Additional file 7: Data S6**. Significant functions differentiating the rumen of L-FCR from H-FCR cattle.

**Additional file 8: Data S7**. Targeted functional profiling of GH11 and GH45 families.

## Data Availability

The dataset analyzed in this study is publicly available in MG-RAST under the study accession number mgp19999. Other relevant data supporting the findings of the study are available in the Supplementary Material files (Data 1-7). Additional data related to this research may be requested from the authors.
