# Supplementary material for: Accelerated discovery of novel glycoside hydrolases using targeted functional profiling and selective pressure on the rumen microbiome
Source: Microbiome. 2021 Nov 23;9:229. doi: 10.1186/s40168-021-01147-1 (PMC8609826; doi:10.1186/s40168-021-01147-1)
Supplement: Supplementary file 2 — Additional file 1: Fig. S1. Feed conversion ratio (FCR) and average daily gain (ADG) in bulls fed forage diets and divided into two groups of feed efficiency: 1) efficient or low feed conversion rate (L-FCR) and 2) inefficient or high-FCR (H-FCR). Fig. S2. Top 10 most abundant phyla detected in the rumen metatranscriptome of cattle fed forage-based diets. Fig. S3. Top 10 most abundant Level 2 functions detected in the rumen metatranscriptome of cattle fed forage-based diets. Fig. S4. Analysis of multivariate homogeneity of group dispersions between H-FCR and L-FCR. Fig. S5. Protozoa and fungi species identified in the rumen metatranscriptome according to the feed efficiency groups. Fig. S6. (A) Homology model of xylanases identified in the rumen microbiome aligns well with crystal structures of xylanases of the GH11 family. Fig. S7. (A) Homology model of xylanases and endoglucanases identified in the rumen microbiome bound to the predicted ligands. Table S1. Ingredient and chemical composition of the forage diet used in this study. Table S2. Details of the homology models predicted by I-TASSER. Amino acid sequences of the ten most abundant GH11 and GH45 enzymes identified in the rumen metatranscriptome. Table S3. Details of the homology models predicted by I-TASSER [3, 4]. [file 40168_2021_1147_MOESM2_ESM.docx]

Supplementary Materials for

**Accelerated discovery of novel glycoside hydrolases using targeted functional profiling and selective pressure on the rumen microbiome**

Andre L. A. Neves, Jiangkun Yu, Yutaka Suzuki, Marisol Baez-Magana, Elena Arutyunova, Eóin O’Hara, Tim McAllister, Kim H. Ominski, M. Joanne Lemieux and Le Luo Guan^*^

^*^Corresponding author: Leluo Guan. E-mail: [lguan@ualberta.ca](mailto:lguan@ualberta.ca)

Short title: Novel enzyme discovery from the rumen microbiome

**This file includes:**

Fig. S1. Feed conversion ratio (FCR) and average daily gain (ADG) in bulls fed forage diets and divided into two groups of feed efficiency: 1) efficient or low feed conversion rate (L-FCR) and 2) inefficient or high-FCR (H-FCR).

Fig. S2. Top 10 most abundant phyla detected in the rumen metatranscriptome of cattle fed forage-based diets.

Fig. S3. Top 10 most abundant Level 2 functions detected in the rumen metatranscriptome of cattle fed forage-based diets.

Fig. S4. Analysis of multivariate homogeneity of group dispersions between H-FCR and L-FCR.

Fig. S5. Protozoa and fungi species identified in the rumen metatranscriptome according to the feed efficiency groups.

Fig. S6. (A) Homology model of xylanases identified in the rumen microbiome aligns well with crystal structures of xylanases of the GH11 family.

Fig. S7. (A) Homology model of xylanases and endoglucanases identified in the rumen microbiome bound to the predicted ligands.

Table S1. Ingredient and chemical composition of the forage diet used in this study.

Table S2. Details of the homology models predicted by I-TASSER.

Amino acid sequences of the ten most abundant GH11 and GH45 enzymes identified in the rumen metatranscriptome.

Legends for data S1 to S7 (including the sample abbreviations used in this study)
References (1-4)

**Fig. S1.** Feed conversion ratio (FCR) and average daily gain (ADG) in bulls fed forage diets and divided into two groups of feed efficiency: 1) efficient or low feed conversion rate (L-FCR) and 2) inefficient or high-FCR (H-FCR). *** means *P* < 0.01 determined by Welch two sample t-test. Estimates (± standard errors) of FCR for L-FCR: 5.7 ± 0.35 kg DMI /kg gain; FCR for H-FCR: 7.3 ± 1.0 kg DMI /kg gain; ADG for L-FCR: 1.6 ± 0.22 kg/day; and ADG for H-FCR: 1.3 ± 0.24 kg/day.

**Fig. S2.** Top 10 most abundant phyla detected in the rumen metatranscriptome of cattle fed forage-based diets according to a bioinformatic pipeline developed by our group [1].

**Fig. S3.** Top 10 most abundant Level 2 functions detected in the rumen metatranscriptome of cattle fed forage-based diets using MG-RAST [2].

**Fig. S4.** Analysis of multivariate homogeneity of group dispersions between High-FCR and Low-FCR groups. Bray–Curtis distances were calculated between samples collected over four-time points (0 – B1, 80 – B2, 100 – B3, and 180 days – B4) and the models were set with 99 permutations to obtain the multivariate dispersions. Tukey's Honest Significant Differences (*P* < 0.05) was used to determine significance between groups.

**Fig. S5.** Protozoa and fungi species identified in the rumen metatranscriptome according to the feed efficiency groups.

**Fig. S6.** (A) Homology model of the ten most abundant xylanases (green) identified in the rumen microbiome aligns well with crystal structures of xylanases of the GH11 family (yellow). (B) Homology model of the three endoglucanases (green) identified in the rumen microbiome aligns well with crystal structures of endoglucanases of the GH45 family (yellow). See Supplementary Table 2 for more details of the homology models.

**Fig. S7.** (A**)** Homology model of the ten most abundant xylanases (green) identified in the rumen microbiome bound to the predicted ligands. (B) Homology model of the three endoglucanases (green) identified in the rumen microbiome bound to the predicted ligands (white). See Supplementary Table 2 for more details of the homology models.

**Table S1.** Experimental design included 4 pens of sixty bulls fed either forage or grain-based diets over two periods^1^

| Pens | Period 1  (0 - 80 Days) | Period 2  (100-180 Days) |
| --- | --- | --- |
| 1 (n= 15 bulls)^2^ | Forage | Forage |
| 2 (n= 15 bulls) | Forage | Grain |
| 3 (n= 15 bulls) | Grain | Forage |
| 4 (n= 15 bulls) | Grain | Grain |

^1^Sixty purebred Angus bulls were randomly assigned into four pens (n = 15 per pen) and fed forage or grain diets over two experimental periods (Period 1 and 2, each with an 80-day duration; the adaptation period between the feeding periods was 20 days). In the first feeding period (FP1), two pens (pen 1 and pen 2) were fed forage-based diets and the remaining two pens (pen 3 and pen 4) were fed a grain-based diet on an *ad libitum* basis. Following FP1, the animals in pen 2 were switched from a forage-based to a grain-based diet, and the animals in pen 3 were switched from grain to forage. Bulls in pens 1 and 4 were exclusively fed forage and grain diets, respectively, in both FPs.

^2^For the current study, 15 purebred Angus Bulls (mean age of 249 ± 22 days; average body weight of 313.9 ± 32 kg at the outset of the experiment) were selected from pen 1 because they were exclusively fed forage diets, which was used as a selection pressure factor to enrich the rumen with fiber degrading microbes and their enzymes. Three animals were removed from our study because they did not maintain the same FCR ranking throughout the experiment.

| **Ingredient composition, %, as-fed basis** |  |
| --- | --- |
| Alfalfa Hay | 17.9 |
| Corn Silage | 81.7 |
| Limestone | 0.2 |
| Mineral | 0.1 |
| Salt | 0.1 |
| **Chemical composition, DM basis** |  |
| Dry Matter, % | 50.7 |
| Acid Detergent Fiber, % | 21.78 |
| Neutral Detergent Fiber, % | 40.89 |
| Total Digestible Nutrients, % | 73.51 |
| Starch, % | 21.6 |
| Metabolizable Energy, MJ kg^-1^ | 11.10 |
| Crude Protein, % | 13.1 |
| Calcium, % | 0.89 |
| Phosphorus, % | 0.45 |
| Magnesium, % | 0.42 |
| Potassium, % | 2.16 |

**Table S2.** Ingredient and chemical composition of the forage diet used in this study

**Table S3.** Details of the homology models predicted by I-TASSER [3, 4].

| **Family** | **Protein_Number** | **Uniprot ID** | **PDB_Homolog** | **Ligand_PDB** | **Ligand_Name** | **C-Score** | **Estimated TM-score** | **Estimated RMSD** |
| --- | --- | --- | --- | --- | --- | --- | --- | --- |
| GH11 | 1 | G5DDC1 | [1h4hB](http://www.rcsb.org/pdb/explore/explore.do?structureId=1h4h) | [1c5iA](http://zhanglab.ccmb.med.umich.edu/BioLiP/qsearch_pdb.cgi?pdbid=1c5i) | 1,2-deoxy-2-fluoro-xylapyranose | 1.67 | 0.95±0.05 | 0.47 Å |
| GH11 | 2 | A0A0A8LFJ4 | [1ynaA](http://www.rcsb.org/pdb/explore/explore.do?structureId=1yna) | [1c5iA](http://zhanglab.ccmb.med.umich.edu/BioLiP/qsearch_pdb.cgi?pdbid=1c5i) | 1,2-deoxy-2-fluoro-xylopyranose | 0.84 | 0.83±0.08 | 0.81 Å |
| GH11 | 3 | A0A0F7TQZ8 | [2vgdA](http://www.rcsb.org/pdb/explore/explore.do?structureId=2vgd) | [1c5iA](http://zhanglab.ccmb.med.umich.edu/BioLiP/qsearch_pdb.cgi?pdbid=1c5i) | 1,2-deoxy-2-fluoro-xylopyranose | 1.34 | 0.90±0.06 | 0.70 Å |
| GH11 | 4 | A0A165DD01 | [2vgdA](http://www.rcsb.org/pdb/explore/explore.do?structureId=2vgd) | [3zseA](http://zhanglab.ccmb.med.umich.edu/BioLiP/qsearch_pdb.cgi?pdbid=3zse) | 1,2-deoxy-2-fluoro-4-O-Beta-D-xylopyranosyl-Beta-D-Xylopyranose | 0.7 | 0.80±0.09 | 0.55 Å |
| GH11 | 5 | G1X0J7 | [1xndA](http://www.rcsb.org/pdb/explore/explore.do?structureId=1xnd) | [3zseA](http://zhanglab.ccmb.med.umich.edu/BioLiP/qsearch_pdb.cgi?pdbid=3zse) | 1,2-deoxy-2-fluoro-4-O-Beta-D-xylopyranosyl-Beta-D-Xylopyranose | 1.69 | 0.95±0.05 | 0.41 Å |
| GH11 | 6 | A0A0A8LF36 | [1xndA](http://www.rcsb.org/pdb/explore/explore.do?structureId=1xnd) | [1c5iA](http://zhanglab.ccmb.med.umich.edu/BioLiP/qsearch_pdb.cgi?pdbid=1c5i) | 1,2-deoxy-2-fluoro-xylopyranose | 1.56 | 0.93±0.06 | 0.47 Å |
| GH11 | 7 | A0A2P2HMK2 | [1xndA](http://www.rcsb.org/pdb/explore/explore.do?structureId=1xnd) | [1c5iA](http://zhanglab.ccmb.med.umich.edu/BioLiP/qsearch_pdb.cgi?pdbid=1c5i) | 1,2-deoxy-2-fluoro-xylopyranose | 1.67 | 0.95±0.05 | 0.43 Å |
| GH11 | 8 | A0A135LSW2 | [1xndA](http://www.rcsb.org/pdb/explore/explore.do?structureId=1xnd) | [1c5iA](http://zhanglab.ccmb.med.umich.edu/BioLiP/qsearch_pdb.cgi?pdbid=1c5i) | 1,2-deoxy-2-fluoro-xylopyranose | 1.69 | 0.95±0.05 | 0.52 Å |
| GH11 | 9 | A0A0N1H0F9 | [2vgdA](http://www.rcsb.org/pdb/explore/explore.do?structureId=3b5l) | [1c5iA](http://zhanglab.ccmb.med.umich.edu/BioLiP/qsearch_pdb.cgi?pdbid=1c5i) | 1,2-deoxy-2-fluoro-xylopyranose | 1.69 | 0.95±0.05 | 0.34 Å |
| GH11 | 10 | A0A0A1CM37 | [1enxA](http://www.rcsb.org/pdb/explore/explore.do?structureId=1enx) | [1bvvA](http://zhanglab.ccmb.med.umich.edu/BioLiP/qsearch_pdb.cgi?pdbid=1bvv) | 1,2-deoxy-2-fluoro-xylopyranose | 1.23 | 0.88±0.07 | 0.48 Å |
| GH45 | 11 | A0A094CQ25 | [3engA](http://www.rcsb.org/pdb/explore/explore.do?structureId=3eng) | [4engA](http://zhanglab.ccmb.med.umich.edu/BioLiP/qsearch_pdb.cgi?pdbid=4eng) | Cellotriose | 2.0 | 0.99±0.04 | 0.57 Å |
| GH45 | 12 | C9RR38 | [5h4uA](http://www.rcsb.org/pdb/explore/explore.do?structureId=5h4u) | [4engA](http://zhanglab.ccmb.med.umich.edu/BioLiP/qsearch_pdb.cgi?pdbid=4eng) | Cellotriose | -0.02 | 0.71±0.12 | 0.89 Å |
| GH45 | 13 | D9SBI1 | [5h4uA](http://www.rcsb.org/pdb/explore/explore.do?structureId=5h4u) | [4engA](http://zhanglab.ccmb.med.umich.edu/BioLiP/qsearch_pdb.cgi?pdbid=4eng) | Cellotriose | 1.57 | 0.93±0.06 | 0.75 Å |

**Amino acid sequences of the ten most abundant GH11 enzymes identified in the rumen metatranscriptome**

>Xylanase1

TLTNNASGKIDGLDYELWKDYGNTSMNLYGGGKFDCSWSSINNALFRIGKKWDCTKTWDQLGSIVVKYGVDYQPNGNSYLCVYGWTRSPLIEYYIVESWGTWRPPGGTSRGKVTVDGGTYDVYVTDRINQPSIDGDTTFKQFWSVRTEKKTSGSISVDKHFSAWTSMGLKLGLMYEASLNVEGYQSSGKAAIYQNDVIGG

>Xylanase2

LTASSTGTNGGYYYSFWTDGGAQVTYTNDAGGEYALTWSGNGNFVGGKGWNPGSAQDVSFSGSYNPDGNSYLSVYGWTTSPLAEYYILEDFGTYNPASSLTYKGSLTSDGSTYDVYEGQRVNEPSIQGTATFNQYWSIRSSKRSSGTVTTANHFSAWAGLGLAMGCLHYQIVATEGYDSSGSSTITAGSAGSGSSGGGGG

>Xylanase3

TITTSQTGTNNGYYYSFWTNGAGTVDYTNGAGGQYSVSWANQNGGDFTCGKGWNPGSAQAISFSGTFNPNGNAYLAVYGWTTSPLVEYYIMESYGDYNPGNSMTHKGTVTSDGATYDIYEHQQVNQPSIQGTATFNQYWSIRQSKRTSGTVTTANHFNAWAALGMNMGAHNYQILSTEGYESSGSSSITVSAGSSSGG

>Xylanase4

MDVEPREPAELAERQTITTSQTGTNNGYYYQLWTAGTGTVDYTNGAAGQYSVKWSKVGDFVAGKGWSTGSARSITFSASSLSLGTGYLSVYGWSTNPLVEYYIVENWAGYNPSSGGTYKGTLTSDGGTYNIYEDTRTNEPSIEGTATFNQYWSVRQSPRTSGTVTTGNHFNAWASHGMPLGTFNYQIVATEAFSSSSGSATVTVS

>Xylanase5

NGYFYSWWSDGGGSAQYTMGEGSKYSVTWRNTGNFVGGKGWNPGNGRTINYGGYFAPSGNGYLAVYGWTRNPLVEYYVVESYGTYNPSSGGTYKGTVNTDGGTYNLYQSTRYNQPSIDGTQTFQQYWSVRTSKRVGGSVNMQAHFNAWANAGMRLGNHYYQIVATEGYQSSGSSEIYV

>Xylanase6

TGTSNGFYYSWWTDGAAQATYTNGGGGQYSLNWSGNNGNLVGGKGWNPGTNGRVINYSGNYQPNGNSYLSVYGWTRSPLIEYYIVESYGSFNPSSAAAKKGSVSCDGATYDILTTTRVNAPSIDGTQTFQQFWSVRNPKKSPGGAISGSVNTGCHFNAWRNLGMNLGTSWNYQIVATEGFKSSGSASITVS

>Xylanase7

TGECNGYHYSFWTDGGGKVEYNNEKNGEYSVSWENCGDFTSGKGWSTGSARNIHFAGDFNPSGNAYLAVYGWTRGPLVEYYILENYGTYNPGPSLTYKGSFTSDGSVYDIYTHQQVDQPSIDGPKKTFMQYWSIRRNKRSDGTVTTANHFNAWASHGMNLGAHDYQILSTEGFASSGHAHMSVW

>Xylanase8

ITQNERGTNGGYFYSFWTNGGGSVSYNNGNAGQYSVNWKNCGSFTSGKGWATGSARNINFSGNFNPSGNAYLAVYGWTKGPLVEYYIMENYGEYNPGGSMTFKGTVTSDGSVYDIYKHTQVNQPSIISDSSTFDQYWSIRRNKRSSGTVTTGNHFNAWAKLGMGLGSHDYQIVNTEGYQSSGSATITVS

>Xylanase9

TTNQTGTNNGYYYSFWTDTQGSVSMNLGSGGNYSTSWSNTGNFVAGKGWSTGGRRSVTYSGTFTPSGNGYLSLYGWTSNPLVEYYIVDNWGTYRPTGTYMGTVSSDGGTYDIYKTTRYNAPSVEGTRTFDQYWSVRQTKRTGGTITTGNHFDAWARAGMPLGSFNYYMILATEGYQSSGNANITVGDAG

>Xylanase10

NCYLSVYGWTTNPLVEYYIVESFGSYDPSSAAQSVGSVQSDGGTYTILQTTRYNQPSIVGTATFPQYWSVR

**Amino acid sequences of the GH45 enzymes identified in the rumen metatranscriptome**

>Endoglucanase1

GNGKTTRYWDCCKPSCAWSGKASFKTGPVQSCDKGDNVLADADTKSACDNGGPAFMCSDESPWAVSDSLAYGFAAVSISGGTEASWCCACYELTFTSGPVSGKKMVVQATNTGGDLGQNHFDIGMPGGGFGLFNACTPQYGTPSTGWGNQYGGLTSRSQCDAFPQALKAGCYWRFDWFQNADNPSVSFKSVACPLALTNKSGCVRSDDTPT

>Endoglucanase2

GQQGFATRYWDCCMPHCSWPEHGGAAKTCDAKGKTPISNTNGSICSGGQGTTCTSQIPIIVSDKLAYAFAATPGNDATCGKCFALTFTGTGKYETKANHQALKGKTLVVMASNIGYDVQGGQFDIMIPGGGFGAFNGCSQMGWNIPQNTTTYGGLLSDCEKEVGYNGNLLTLRKECLTKKCNSAFASDTQAKEGCLFLATWMEAAGNPNHTYKEVECPAALKAKF

>Endoglucanase3

SGWNTRYWDACKAHCSQTSLDGAEGKPVITSQEEYETAREIPTFNQEADPASGGGFTCTDMAPVAVNDTLAYAFVAGSDATTSCGKCFHLQYDHKALKGKHIIVMASNIGHDVKPGQFDLMVPGGGPGIFNALQLQITKPGIEWGATYGGFLTYCQCDAAFGDSNYPNLLRGCHWFADWFMAADNPTYQWEEVECPQYLVDKYETTISRS

**Legends for Sample IDs and Supplementary Data:**

Sample IDs:

In the Supplementary Data, the IDs of rumen samples (n = 48) were represented by high- and low-feed conversion ratio bulls (H- and L-FCR, respectively), sequenced over four time points (D1 – 0 day; D2 – 80 Day; D3 – 100 Day; and D4 – 180 Days).

For example:

H-FCR bulls (n= 24 samples): "17ZD1H","17ZD2H","17ZD3H","17ZD4H", "1ZD1H", "1ZD2H", "1ZD3H", "1ZD4H","2094ZD1H", "2094ZD2H", "2094ZD3H", "2094ZD4H", "29ZD1H", "29ZD2H", "29ZD3H", "29ZD4H", "77D1H", "77D2H", "77D3H", "77D4H", "8ZD1H", "8ZD2H", "8ZD3H", "8ZD4H".

L-FCR bulls (n= 24 samples): "126D1L", "126D2L", "126D3L", "126D4L", "162ZD1L", "162ZD2L", "162ZD3L", "162ZD4L", "20LZD1L", "20LZD2L", "20LZD3L", "20LZD4L", "2ZD1L", "2ZD2L", "2ZD3L", "2ZD4L", "60ZD1L", "60ZD2L", "60ZD3L", "60ZD4L", "91ZD1L", "91ZD2L", "91ZD3L", "91ZD4L".

Data S1 to S7:

Data S1. FCR ranking of the experimental animals.

Data S2. Assembly statistics of the samples investigated in this study.

Data S3. Bacterial composition (phyla, family, genera, and species) detected in the rumen metatranscriptome of forage-fed bulls.

Data S4. Microbial functions (levels 1, 2, 3 and 4) detected in the rumen metatranscriptome of forage-fed bulls.

Data S5. CAZyme families identified in this study.

Data S6. Significant functions differentiating the rumen of L-FCR from H-FCR cattle.

Data S7. Targeted functional profiling of the GH11 and GH45 families.

**References**

1. Neves ALA, Li F, Ghoshal B, McAllister T, Guan LL: **Enhancing the resolution of rumen microbial classification from metatranscriptomic data using Kraken and Mothur**. *Frontiers in Microbiology* 2017, **8**(2445).

2. Meyer F, Paarmann D, D'Souza M, Olson R, Glass E, Kubal M, Paczian T, Rodriguez A, Stevens R, Wilke A *et al*: **The metagenomics RAST server – a public resource for the automatic phylogenetic and functional analysis of metagenomes**. *BMC Bioinformatics* 2008, **9**(1):386.

3. Yang J, Yan R, Roy A, Xu D, Poisson J, Zhang Y: **The I-TASSER Suite: protein structure and function prediction**. *Nature Methods* 2014, **12**:7.

4. Zhang Y: **I-TASSER server for protein 3D structure prediction**. *BMC Bioinformatics* 2008, **9**(1):40.
